# Differential responses of hepatopancreas transcriptome between fast and slow growth in giant freshwater prawns (*Macrobrachium rosenbergii*) fed a plant-based diet

**DOI:** 10.1038/s41598-024-54349-6

**Published:** 2024-02-29

**Authors:** Khanakorn Phonsiri, Rapeepat Mavichak, Stephane Panserat, Surintorn Boonanuntanasarn

**Affiliations:** 1 School of Animal Technology and Innovation, Institute of Agricultural Technology, Suranaree University of Technology, 111 University Avenue, Muang, Nakhon Ratchasima, 30000 Thailand; 2 Aquatic Animal Health Research Center, Charoen Pokphand Co. Ltd., Rama 2 Rd., Km 41.5, Bangtorat, Muang Samutsakorn, Samutsakorn, 74000 Thailand; 3 INRAE, Université de Pau et des Pays de l’Adour, E2S UPPA, NuMéA, Saint-Pée-Sur-Nivelle, France

**Keywords:** *Marcrobrachium rosenbergii*, Plant-based diet, RNA sequencing, Growth, Hepatopancreas, Biotechnology, Molecular biology

## Abstract

Efficient utilisation of plant-based diets in the giant freshwater prawn, *Marcrobrachium rosenbergii*, varies according to individual, suggesting that it might be associated with differences in physiological and metabolic responses. Therefore, we aimed to investigate the individual differences in the growth response of shrimp fed to a soybean-based diet (SBM). Two hundred shrimp were fed SBM for 90 days, and specific growth rate (SGR) was determined individually. Fast- and slow-growing shrimp (F-shrimp vs. S-shrimp), with the highest and lowest 5% SGRs, respectively, were sampled to determine haemolymph chemistry and carcass composition. The hepatopancreas of these shrimps were used for transcriptome analysis through RNA sequencing (RNA-Seq). The results showed no significant differences in haemolymph chemistry parameters. In terms of carcass proximate composition, F-shrimp exhibited higher protein composition than did S-shrimp, suggesting that F-shrimp have higher protein anabolism. Using RNA-seq and real-time reverse transcription polymerase chain reaction (qRT-PCR), the expression levels of several genes encoding physiologic and metabolic enzymes were found to be upregulated in F-shrimp compared to in S-shrimp, suggesting that these enzymes/proteins mediated the efficient use of SBM-based diets for growth promotion in shrimp. Various DEGs associated with the immune system were observed, indicating a difference in immune processes between F- and S-shrimp. The expression of several housekeeping genes was found to be upregulated in S-shrimp. Collectively, the upregulated expression of several enzymes associated with physiological and/or metabolic processes and increased protein anabolism may be attributed to the efficient use of SBM for maximal growth in shrimp.

## Introduction

Global aquaculture has sustainably contributed to food and nutrition security in the global consumption of animal-source proteins. Aquaculture has become a rapidly growing food production sector worldwide. For instance, during 2010–2018, annual production of aquaculture increased from 59.7 to 82.1 million tonnes. Among the aquatic species produced, during 2010–2018, annual production of shrimp rapidly increased from 5478.8 to 9386.5 thousand tonnes^1^. In addition, over the next decade, it has been estimated that aquaculture and shrimp production will continue to expand. Aquaculture is the only agricultural sector in animal production that uses fishmeal in the diets of the organisms produced. Owing to the dependence of the aquafeed industry on fishmeal use, particularly for carnivorous species, including shrimp, the expansion of aquaculture has led to a growing share of global fishmeal utilisation^2,3^. A limited supply of fishmeal in the future might impede the continuous expansion of aquaculture and/or result in high feed costs. Fishmeal replacements containing alternative protein sources for aquafeeds include plant-based protein ingredients, terrestrial animal by-products, single-cell proteins, and insect meals^4–6^. Using plant feedstuffs for sustainable aquaculture represents a challenge. Indeed, numerous studies have demonstrated the effects of total and partial replacements of fishmeal with plant ingredients in shrimp^9,10^.

Among plant feedstuffs, soybean products have been used as an alternative protein source to satisfy protein requirements in shrimp owing to high protein content, amino acid profile, and a certain supply of soybean meal (SBM)^7–10^. The negative responses of shrimp to SBM-incorporated diets may be attributed to the presence of phytochemicals, including anti-nutritional factors, phytoestrogens, dietary fibres, enzyme inhibitors, and high carbohydrate content^11^. The incorporation rate of SBM in shrimp diet with no negative impact on shrimp performance and/or health status varies depending on the species, growth phase, and duration of feeding^9,10,12–14^. The growth performance of fish fed plant-based diets also varies according to the fish family/individual. For example, variations in growth performance were observed in zebrafish and Atlantic salmon fed plant protein-based and plant lipid-based diets, respectively^15,16^. Recently, a nutrigenetic approach revealed genetic variants, such as single nucleotide polymorphisms (SNP), in growth traits related to the efficacy of specific nutrient utilisation in rainbow trout and zebrafish^17,18^. In addition, variations in growth and/or nutritional status among individuals might be attributed to differences in physiological and metabolic responses to specific nutrients^6,10,13^.

High-throughput RNA sequencing (RNA-seq) technology has proved to be a useful tool for the elucidation of gene expression in terms of global networks and evaluating differences in gene expression in particular tissues and circumstances. Consequently, in the nutrigenetics approach, RNA-seq information, including de novo transcriptome assembly, functional annotation, and enrichment analysis, enables the rapid exploration of physiological and metabolic responses to specific nutrients in fish and shrimp^19–22^. Among shrimp producers, the giant freshwater prawn (*Macrobrachium rosenbergii*), which is the largest freshwater prawn in the world, is an economically important species in global aquaculture, especially in Asia and America. Generally, giant freshwater prawn larvae grow in brackish water through the post-larval stage and subsequently live in freshwater adjacent brackish water areas^23^. Although not as high as marine shrimp production, the global production of freshwater prawns has increased. In farming systems, giant freshwater prawns can utilise fishmeal-free or plant-based diets, such as SBM-based diets^7^. The efficient use of plant-based diets varies according to shrimp individuals, and differences in the efficient intake of plant-based diets may be related to differences in metabolic responses. Therefore, to improve the efficiency of plant-based protein diets in promoting growth in shrimps, intensive investigation of physiological and metabolic changes according to dietary soybean meal inclusion at high levels is required^6,12^.

Therefore, herein, we aimed to investigate individual differences in growth responses of shrimp to SBM-based diets. Using RNA-seq, we performed de novo transcriptome assembly, functional annotation, and enrichment analysis in shrimp hepatopancreas, which is the primary tissue responsible for metabolic and physiological processes. In addition, we identified differentially expressed genes (DEGs) between fast- and slow-growing shrimp. Furthermore, the expression levels of several DEGs crucial to metabolic pathways were validated using real-time reverse transcription polymerase chain reaction (qRT-PCR).

## Materials and methods

### Experimental processes, diet, and shrimp rearing

All experimental procedures involving shrimps were performed in accordance with relevant guidelines and regulations of ARRIVE (Animal Research: Reporting of In Vivo Experiments) which were approved by the Ethics Committee of the Suranaree University of Technology Animal Care and Use Committee (approval no. U1-08913-2563). The freshwater shrimp generally showed sexual dimorphism in growth, and male fish grow faster than female^24^. To avoid the confounding effect associated with sex dimorphism, only male shrimps were selected for this study. Two hundred freshwater shrimps (*M. resenbergii*) (37–59 g) were collected from 22 commercial farms in central Thailand. Note that since experimental shrimp were obtained from earthen pond, there were high variation in initial body weight. During a week of adaptation to the experimental pond, shrimps were maintained in a cement pond (W5 × L10 × H0.8 m) at the University Farm, Suranaree University of Technology, Nakhon Ratchasima, Thailand. Shrimps were fed with a commercial diet (36% crude protein (CP) + 6% crude fat (CF)) at 3% body weight twice daily. After adaptation to the experimental pond, shrimps were weighed and randomly distributed to each partition (W40 × L40 × H60 cm) for individual rearing in an experimental pond (cement pond; W5 × L10 × H0.8 m) at a stocking density of 4 shrimps/m^2^. For acclimatisation to the experimental diet (10 days), shrimps were fed a diet that was gradually changed from commercial diet to experimental diet. Table 1 shows the composition of the experimental diets and proximate composition, including moisture, CP, CF, crude fibre, and ash, which were analysed according to the standard method described in the guidelines of the Association of Official Analytical Chemists^25^. The experimental period of 90 days started when shrimps completely accepted each treatment diet. Dead shrimps were recorded to determine the survival rate. Through the end of the experimental period, the survival rate was 60.57% which were acceptable for shrimp culture condition. The final weight of each shrimp individual was determined to calculate specific growth rate, and growth performance was also determined.

**Table 1:** Ingredient composition and proximate analysis of the experimental diet.

Ingredient (g kg^–1^)	
Soybean meal	427
Wheat gluten meal	140
Rice bran	100
Wheat flour	170
Shrimp head meal	40
Fish oil/soybean oil	40
Methionine	2
Lecithin	10
Cholesterol	10
Astaxantine	1
Sodium propionate	10
Dicalcium phosphate	10
Mineral and vitamin premix^1^	40
Proximate analyses (%)	
Dry matter	93.62 ± 0.30
Crude protein	35.86 ± 0.17
Crude lipid	6.19 ± 0.03
Crude fiber	3.60 ± 0.37
Ash	9.18 ± 0.03
Nitrogen free extract	38.65 ± 0.76
Gross energy (kcal g^–1^)	4107.21 ± 30.75

^1^Vitamin and trace mineral mix provided the following (IU kg^−1^ or g kg^−1^diet): vitamin A, 15,000 IU; vitamin D3, 3,000 IU; vitamin E, 300 IU; vitamin K, 0.024 g; vitamin B1, 0.0075 g; vitamin B2, 0.036 g; vitamin B6, 0.0225 g; vitamin B12, 0.0002 g; vitamin C, 3 g; pantothenic acid, 0.09 g; niacin, 0.0645 g; folic acid, 0.009 g; inositol, 0.75 g; biotin, 0.0008 g; selenium, 0.0009 g; iron, 0.6 g; zinc, 0.96 g; copper, 0.06 g.

Throughout the experimental period, air and water temperatures, in ranges from 25.4 to 28.8 °C and 26.0 to 27.8 °C, respectively, were determined daily. Dissolved oxygen (DO) and pH were recorded weekly using DO and pH meters, respectively, observed to be 4.5 ± 0.2 mg/L (average ± SD) and in ranges of 7.99–8.51, respectively. DO and pH were within acceptable ranges for freshwater shrimp growth.

### Experimental design, shrimp sampling, and haemolymph collection

At the end of the experiment, the specific growth rate (SGR) of shrimp was calculated using the following formula: SGR = 100 × [(ln final body weight − ln initial body weight)/experimental days] which was used to rank individual shrimps. For fast-growing shrimps (F-shrimp) and slow-growing shrimps (S-shrimp), the top 5% (9 shrimps; 3 replicates, 3 shrimps/replication) and the bottom 5% (9 shrimps; 3 replicates, 3 shrimps/replication), respectively, were selected for sampling. At 5 h after feeding, the shrimps were euthanised using ice water. To analyse haemolymph metabolites, haemolymph samples were collected from the haemocoel using a hypodermic syringe and mixed with 10.0% (v/v) of 10% sodium citrate v/v (pH 7.9). Haemolymph was collected after centrifugation at 14,000×*g* for 10 min at 4 °C and stored at – 80 °C until analysis. After bleeding, hepatopancreas was collected and stored at − 80 °C for RNA-seq and qRT-PCR. Muscle tissues were collected and stored at − 80 °C for analysis of proximate nutritive composition.

### Haemolymph metabolite analysis and proximate chemical analysis

Haemolymph metabolites (3 shrimps/replicate), including glucose, triglyceride, cholesterol, total protein, haemolymph urea nitrogen (HUN), alanine transaminase (ALT), and aspartate transaminase (AST), were measured. Plasma glucose levels were quantitatively analysed using Trinder’s method^26^. Plasma triglyceride levels were evaluated using 3-sulfopropyl-*m*-anisidine^27^. Cholesterol levels were quantitatively determined using cholesterol oxidase-phenol + aminophenazone (CHOD-PAP)^28^. Determination of HUN were performed using a modified indophenol colorimetric method^29^. Evaluation of ALT and AST levels were carried out according to the recommendations of the International Federation of Clinical Chemistry^30^. Total protein, Mg, Ca, and P in haemolymph were evaluated using Erba's BIO-LA-TEST^®^ (Erba Lachema s.r.o., Brno, Czech Republic). Proximate chemical analyses, including CP, CF, moisture and ash analyses, were performed according to the guidelines of the Association of Official Analytical Chemists (1990)^25^.

### RNA extraction and sequencing

We performed transcriptomic analysis to compare expression levels of differentially DEGs in hepatopancreas, three replicates each, between F-shrimp and S-shrimp. Each replicate of hepatopancreas RNA was obtained from equal amounts of RNA pooled from three hepatopancreas. Therefore, each replicate of hepatopancreatic RNA was obtained from three shrimp individuals.

Total RNA was extracted from the sampled hepatopancreas (approximately 100 mg) using TRIzol reagent (Invitrogen, Carlsbad, CA, USA) and an RNeasy Mini Kit (Qiagen, Hilden, Germany), according to the manufacturer’s instructions. The quantity of isolated RNA was determined using a NanoPhotometer spectrophotometer (IMPLEN, CA, USA). Agarose gel (1%) electrophoresis was also performed to monitor RNA degradation and contamination. The RNA integrity and quantification were assessed using the RNA Nano 6000 Assay Kit of the Bioanalyzer 2100 system (Agilent Technologies, CA, USA). An RNA obtained with the RNA integrity number (RIN) value > 7.0 was used for further analysis. Three replicates of hepatopancreas RNA samples (1 µg) from F-shrimp and S-shrimp were used for RNA sample preparation. Sequencing libraries were generated using the NEBNext® Ultra TM RNA Library Prep Kit for Illumina® (NEB, USA) as per the manufacturer’s protocols, and index codes were added to attribute sequences to each sample. Briefly, mRNA enrichment was performed using poly T oligo-attached magnetic beads. Fragmentation was performed using divalent cations under elevated temperatures in NEBNext First-Strand Synthesis Reaction Buffer (5X). First-strand cDNA was synthesised from all samples using random hexamer primers and M-MuLV reverse transcriptase. Second-strand cDNA synthesis was performed using DNA Polymerase I and RNase H. In the reaction buffer, dNTPs with dTTP were replaced with dUTP. The remaining overhangs were converted into blunt ends via exonuclease/polymerase activity. After adenylation of the 3’ ends of the DNA fragments, NEBNext adaptor with a hairpin loop structure was ligated for hybridisation preparation. To select cDNA fragments of preferentially 250–300 bp in length, the library fragments were purified using the AMPure XP system (Beckman Coulter, Beverly, USA). Then, 3 µl USER Enzyme (NEB, USA) was used with size-selected, adaptor-ligated cDNA at 37 °C for 15 min, followed by 5 min at 95 °C before PCR. PCR was performed using Phusion High-Fidelity DNA polymerase, Universal PCR primers, and Index (X) Primer. Three libraries were prepared for F-shrimp (F-shrimp 1, F-shrimp 2, and F-shrimp 3) and S-shrimp (S-shrimp 1, S-shrimp 2, and S-shrimp 3). Subsequently, the products were purified (AMPure XP system), and library quality was assessed using the Agilent Bioanalyzer 2100 system. Clustering of the index-coded samples was performed on a cBot Cluster Generation System using the PE Cluster Kit cBot-HS (Illumina), according to the manufacturer’s instructions. All libraries were loaded onto a HiSeq 2500 Sequencing System (Illumina), according to the manufacturer’s protocol, at the Novogene Bioinformatics Institute, Beijing, China.

### Pre-processing, de novo assembly, and read pre-processing

Figure S1 presents the workflow of RNA-seq and differential expression analysis. The original raw sequencing data were converted to sequenced reads using base calling, and 126.52 million bases (Gb) of raw reads were generated using the Illumina HiSeq platform. To obtain clean reads, raw reads were filtered to remove those with adapter sequences, containing poly-N, or of low quality. Base calling and quality assignment were evaluated using the Phred score, as per the following formula: Q_phred_ = − 10 log_10_(*e*). Six sets of clean reads were assembled de novo using the Trinity 2.0.6 software with default parameters (k-mer, 25; minimum length, 200 nucleotides)^31^, and then Corset software was used to perform hierarchical clustering to remove redundancy^32^. Finally, the longest transcript of each cluster was selected as the unigene.

### Transcriptome functional annotation

For transcriptome functional annotation, seven databases were employed including nr (National Center for Biotechnology Information [NCBI] non-redundant protein sequences) and nt (NCBI BLAST 2.2.28 +). Swiss-Prot databases were used with an E-value threshold of 10^–5^ to obtain the top 10 alignment results for annotation of the assembled unigenes. In addition, the transcripts were searched against the Eukaryotic Orthologous Groups (KOG) database, with the significance E-value threshold set at 10^–3^. Unigene annotations were also searched against the Protein Family (Pfam) database using the HMMER 3.0 software package with an E-value threshold of 0.01. For gene ontology (GO) mapping, based on the protein annotation results of the BLAST search and Pfam, Blast2GO 2.5^33^ was used to determine the GO annotation associated with the hits obtained from the BLAST search and Pfam, with a significant E-value threshold of 10^–6^ for presenting biological processes, molecular functions, and cellular components. The KOG classification of the unigenes was analysed. The sequences were also annotated and characterised using Kyoto Encyclopedia of Genes and Genomes (KEGG), with a significant E-value threshold set at 10^–10^. The species distribution of the top BLASTX results matching with the nucleotide database was also analysed.

### Analysis of gene expression levels and differential expression

De novo transcriptome assembly was used as a reference for read mapping. Clean reads were mapped back onto the assembled transcriptome using the Bowtie 2 software, and the read count of each gene from each sample was estimated. The gene expression levels of each sample were estimated using RSEM software^34^. The read count for each gene in each sample was normalised to fragments per kilobase of transcript sequence per million base pairs sequenced (FPKM)^35^. A Venn diagram was generated to present the number of transcripts that were uniquely expressed and co-expressed in F-shrimp and S-shrimp. Differential expression analysis was performed at the unigene level through pairwise comparisons between F-shrimp and S-shrimp using the R package DEGseq^36^. The false discovery rate (FDR) *P*-value was adjusted using the q-value. A q-value < 0.05 and log_2_ fold change > 1 were set as the thresholds for significant DEGs.

### Realtime-RT-PCR analysis

According to RNA sequence information, this study selected several genes that were essential in metabolic and immune processes including crustapain (*cys*), crustin (*crus*), actin cytoplasmic 1 (*actb*), ornithine decarboxylase antizyme-1 (*odc-az*), 6-phosphogluconate dehydrogenase (*6pgd*), tumor necrosis factor alpha-induced protein 8 (*tnfaip8*), coiled-coil and C2 domain-containing protein 1A (*cc2d1a*), furry protein (*fry*), C-type lectin 1 (*clec*), insulin-like androgenic gland hormone binding protein (*iagbp*), signal-induced proliferation-associated 1-like protein 2 isoform X2 (*sipa1l2*), vacuolar protein sorting-associated protein 13A-like isoform X2 (*vps13a*), Rab GTPase-binding effector protein 1 (*rabep1*), and friend leukemia integration 1 transcription factor (*fli1*) to validate differences in expression level between F-shrimp and S-shrimp using qRT-PCR analysis. Table S1 lists the primers and expected sizes of the amplicons. Briefly, using the same RNA samples (2 µg) as those used for the RNA-seq library preparation, first-strand cDNA was conducted using the ImProm-II™ Reverse Transcription System kit (Promega). To determine transcript levels, qRT-PCR amplification (in triplicate) was conducted using a LightCycler® 480 SYBR Green I Master Mix (Roche Applied Science, Indianapolis, IN, USA). Among common internal reference genes for qRT-PCR in shrimps such as elongation factor and actin^37^, the expression of 18 s ribosomal RNA (18S rRNA) was used as an internal reference for data normalisation since its relative expression did not significantly vary between two experimental shrimps. The PCR samples were prepared in a final volume of 10 µL, comprising 5 µL of LightCycler® 480 SYBR Green I Master, 500 nM of each primer, and 1 µL of cDNA template. The PCR reaction was performed at 95 °C for 10 min, followed by 50 reaction cycles of 15 s at 95 °C, 15 s at 60 °C (Table S1), and 20 s at 72 °C. Upon completion of amplification, all samples were subjected to melting curve analysis to distinguish between PCR products. The negative controls used cDNA-template-free samples. To analyse mRNA levels, relative quantification of target gene expression was performed using the Roche Applied Science E-method. The data were analysed using the comparative cycle threshold method. The PCR efficiency was measured using the slope of a standard curve constructed using serial dilutions of cDNA. In all analyses, the PCR efficiency values ranged between 1.8 and 2.0.

### Statistical analysis

All data were analysed using SPSS for Windows version 16 (SPSS Inc., Chicago, IL, USA). An independent t-test was used to compare the differences between F-shrimp and S-shrimp. The effects and differences were considered significant at a *P* < 0.05.

### Institutional review board statement

The study protocol was approved by the Ethics Committee of Suranaree University of Technology Animal Care and Use Committee (approval no. U1-08913-2563).

## Results

### Growth, haemolymph metabolites and carcass composition

**Figure 1:**
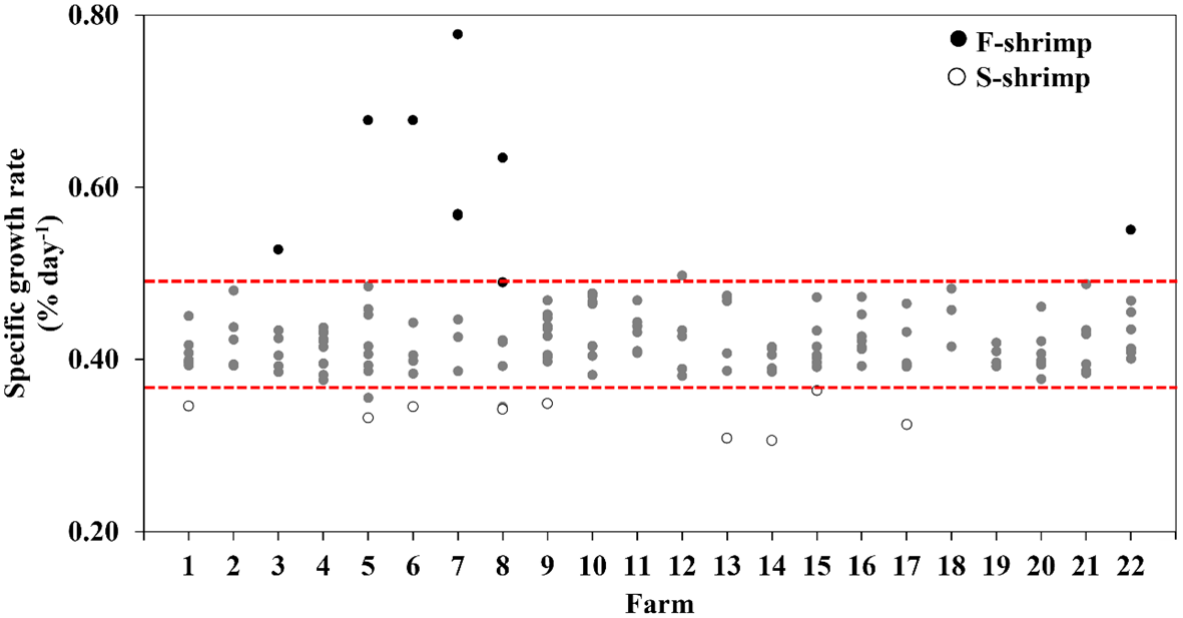
Specific growth rate of experimental fast- and slow- growing shrimps fed an SBM-based diet. Fast-growing shrimp (F-shrimp) and slow-growing shrimp (S-shrimp) were selected from the top and bottom 5% SGR, respectively.

Figure 1 demonstrates the top (F-shrimp) and bottom (S-shrimp) 5% SGR values that were selected in this study. Table 2 showed that weight gain, average daily gain and SGR of F-shrimp were higher than that of S-shrimp (*P* < 0.05). There were no significant differences in haemolymph metabolites, including glucose, triglycerides, cholesterol, total protein, haemolymph urea nitrogen (HUN), ALT, and AST (*P* > 0.05) (Table 3). In addition, haemolymph minerals, including Mg, Ca, and P, appeared to be similar between F-shrimp and S-shrimp (*P* > 0.05) (Table 3). Among the proximate composition of carcasses, F-shrimp had significantly higher protein content than S-shrimp (*P* < 0.05), while there were no significant differences in moisture, lipid, and ash content (*P* > 0.05) (Table 4).

**Table 2:** Growth performance of shrimp fed with SBM-based diet (mean ± SD, n = 9)^1^.

Treatment	Initial body weight (g)	Final body weight (g)	Weight gain (g)	Average daily gain (g day^–1^)	Specific growth rate (% day^–1^)	FCR
F-shrimp	49.28 ± 4.42	86.87 ± 7.32	37.60 ± 4.36	0.40 ± 0.05	0.61 ± 0.05	2.30 ± 0.34
S-shrimp	54.80 ± 0.83	75.16 ± 0.57	20.36 ± 0.77	0.22 ± 0.01	0.34 ± 0.01	2.63 ± 0.08
*p*-value	0.101	0.051	0.018	0.002	0.001	0.161

^1^Independent t-test was used to compare means. See Fig. 1 for selection criteria of experimental shrimps.

Weight gain = final body weight–initial body weight.

Average daily gain = (final body weight–initial body weight)/experimental days.

Specific growth rate = 100 × [(ln final body weight–ln initial body weight)/experimental days].

Feed conversion ratio (FCR) = dry feed fed/wet weight gain.

Note that variation of initial weight of shrimp obtained from commercial farms was high; therefore, no significant difference in final weight was detected.

**Table 3:** Haemolymph chemistry of F-shrimp and S-shrimp (mean ± SD, n = 3)^1^.

	F-shrimp	S-shrimp	*P*-value
Glucose (mmol L^–1^)	1.51 ± 0.37	1.46 ± 0.20	0.848
Triglyceride (mmol L^–1^)	0.31 ± 0.04	0.33 ± 0.06	0.942
Cholesterol (mmol L^–1^)	0.45 ± 0.16	0.46 ± 0.21	0.967
Total protein (mg ml^–1^)	114.56 ± 3.95	112.99 ± 4.92	0.689
HUN (mmol L^–1^)	1.87 ± 0.18	1.72 ± 0.43	0.612
ALT (U L^–1^)	105.63 ± 3.98	106.29 ± 12.76	0.935
AST (U L^–1^)	118.83 ± 4.82	110.20 ± 20.30	0.541
Mg (mmol L^–1^)	21.69 ± 1.13	22.23 ± 1.74	0.674
Ca (mmol L^–1^)	14.67 ± 1.18	15.02 ± 0.96	0.708
P (mmol L^–1^)	1.29 ± 0.24	1.24 ± 0.09	0.716

*HUN* hemolymph urea nitrogen, *ALT* alanine aminotransaminase, *AST* aspartate transaminase.

^1^Independent *t* test was used to compare the haemolymph chemistry parameters between fast-growing shrimp (F-shrimp) and slow-growing shrimp (S-shrimp). There were no significant differences in haemolymph parameters (*P* > 0.05).

**Table 4:** Chemical composition of carcasses of F-shrimp and S-shrimp (mean ± SD, n = 3)^1^.

	F-shrimp	S-shrimp	*P*-value
Moisture (%)	72.66 ± 0.53	73.12 ± 0.47	0.320
Protein (%)	25.12 ± 0.21	23.44 ± 0.09	< 0.001
Lipid (%)	0.49 ± 0.04	0.44 ± 0.01	0.091
Ash (%)	1.68 ± 0.12	1.65 ± 0.12	0.790

^1^Independent t-test was used to compare the chemical composition of carcasses between fast-growing shrimp (F-shrimp) and slow-growing shrimp (S-shrimp). Differences in the mean values were evaluated at $P<0.05$.

### Sequencing assembly and functional annotation of assembled unigenes

Approximately 38 Gb of raw reads, including 126,544,247 raw reads with 125,505,347 clean reads, were obtained (Table 5). Moreover, Phred quality scores of the clean reads at Q20 and Q30 ranged 97.54–97.89% and 92.78–93.54%, respectively. The de novo assembly of each clean read resulted in 44,088 assembled unigenes; 19,518 (44.27%) were able to be annotated in at least one of seven databases (nr, nt, Swiss-Prot, KEGG, GO, KOG, and Pfam) (Table 6). Thereof 1857 unigenes (4.21%) annotated in all seven databases. The experimental unigenes had high level of sequence identity with *Litopenaeus vannamei* (66.1%) and *Hyalella Azteca* (2.7%) (Fig. S2). Figure S3 shows a Venn diagram which demonstrates the distribution of the expressed transcripts of the hepatopancreas between F-shrimp and S-shrimp. There were 18,597 and 23,049 transcripts expressed in the hepatopancreas of F-shrimp and S-shrimp, respectively, and among them, 15,748 were co-expressed between F-shrimp and S-shrimp.

**Table 5:** Summary of the sequencing results.

Sample	Raw reads	Clean reads	Raw bases (G)	Clean bases (G)	Q20 (%)	Q30 (%)	%GC
F-shrimp 1	21,332,950	21,182,938	6.4	6.4	97.55	92.82	43.77
F-shrimp 2	20,824,537	20,678,445	6.2	6.2	97.89	93.54	44.87
F-shrimp 3	21,249,615	21,094,270	6.4	6.3	97.59	92.87	44.27
S-shrimp 1	20,864,536	20,646,939	6.3	6.2	97.59	92.98	44.13
S-shrimp 2	21,872,088	21,704,124	6.6	6.5	97.62	92.96	43.71
S-shrimp 3	20,400,521	20,198,631	6.1	6.1	97.54	92.78	43.42

*F-shrimp* fast-growing shrimp, *S-shrimp* slow-growing shrimp.

**Table 6:** Annotation of RNA-seq results.

Database	Number of unigenes	Percentage (%)
Annotated in NR	15,072	34.18
Annotated in NT	4514	10.13
Annotated in KO	7130	16.17
Annotated in Swiss-Prot	10,625	24.09
Annotated in Pfam	14,272	32.37
Annotated in GO	14,268	32.36
Annotated in KOG	6112	13.86
Annotated in all databases	1857	4.21
Annotated in at least one databases	19,518	44.27
Total unigenes	44,088	100

NCBI non-redundant protein database (nr), NCBI nucleotide sequence database (nt), protein sequence database (Swiss-Prot), Protein Family (Pfam), Gene Ontology (GO), and eukaryotic orthologue groups (KOG) databases were used for alignment of all assembled unigenes.

### Classification of experimental transcripts

**Figure 2:**
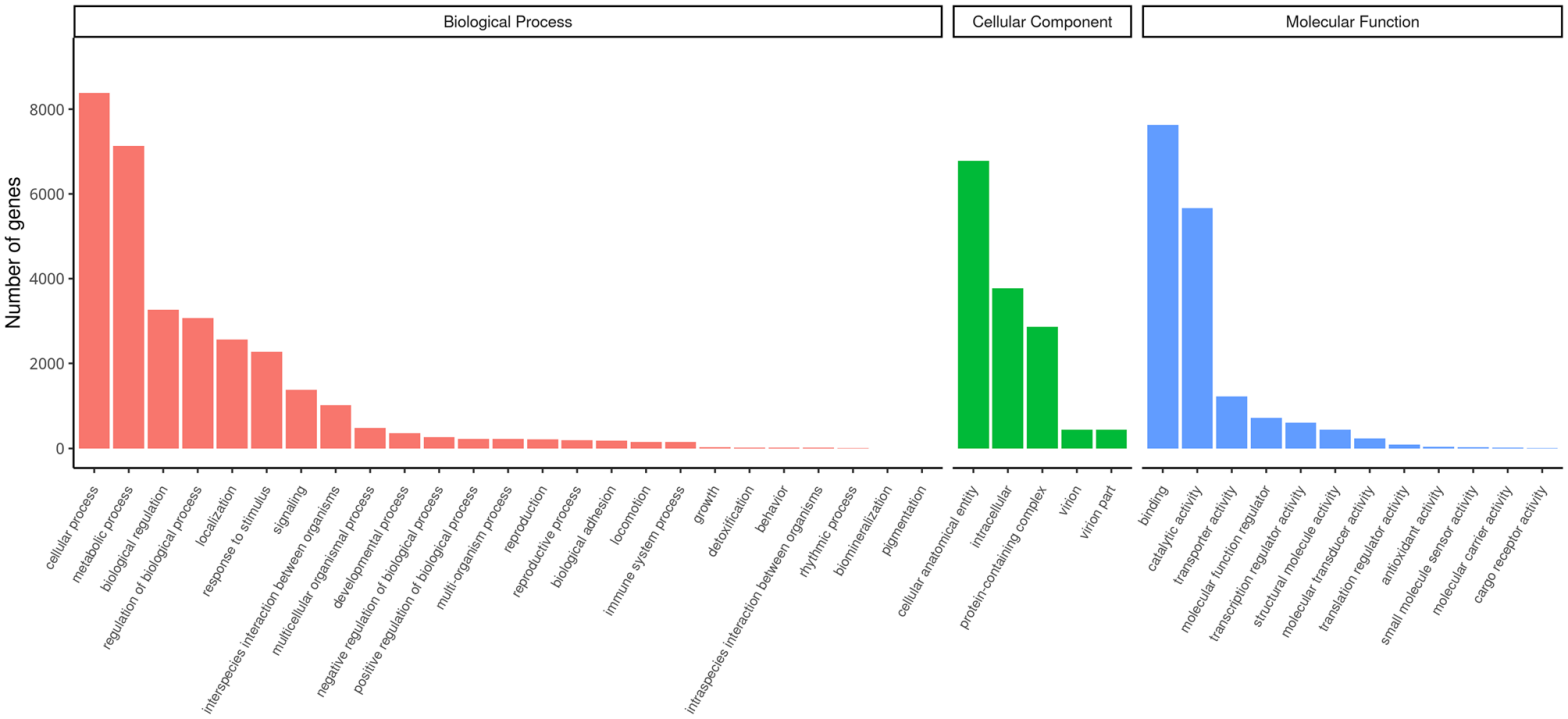
Histogram of gene ontology (GO) classification of all genes that were expressed in hepatopancreas of fast-growing shrimp (F-shrimp) and slow-growing shrimp (S-shrimp). The results are presented in three main categories: biological process, cellular component, and molecular function.

**Figure 3:**
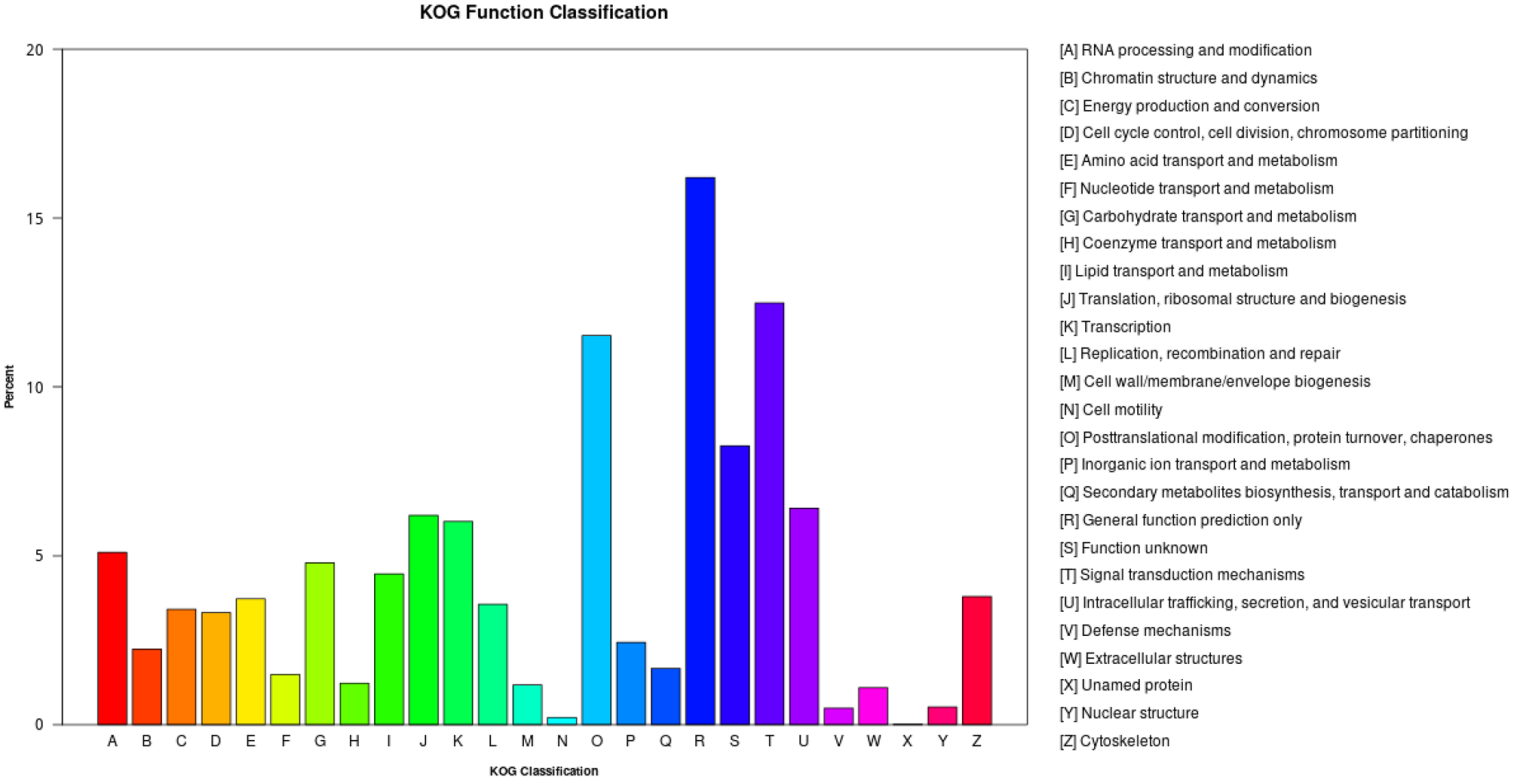
Classification of all genes annotated in KOG that were expressed in hepatopancreas of fast-growing shrimp (F-shrimp) and slow-growing shrimp (S-shrimp). The X-axis represents the classification name of the 26 KOG groups, and the Y-axis represents the percentage of genes in each annotated KOG group.

**Figure 4:**
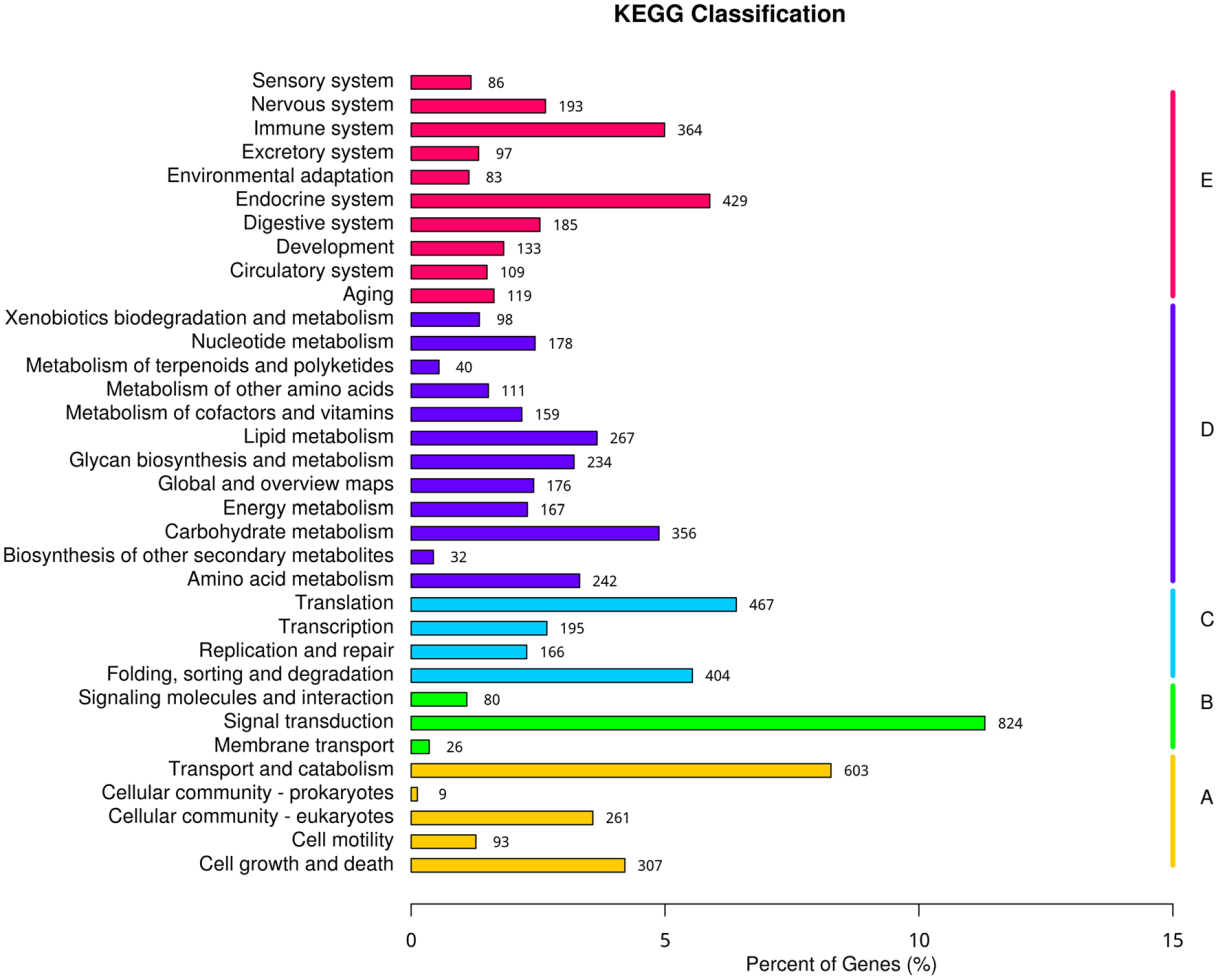
Classification of all genes annotated in Kyoto Encyclopedia of Genes and Genomes (KEGG) pathways that were expressed in hepatopancreas of fast-growing shrimp (F-shrimp) and slow-growing shrimp (S-shrimp). The X-axis represents the percentage of genes in each annotated KEGG pathway, and the Y-axis shows KEGG pathway terms. The KEGG metabolic pathways gene involved in are divided into 5 branches: A: Cellular Processes, B: Environmental Information Processing, C: Genetic Information Processing, D: Metabolism, E: Organismal Systems.

Classification of GO demonstrated that 14,268 unigenes were annotated into 42 functional groups of the three ontologies (Fig. 2). Most unigenes were classified into the “biological process” category, with the majority representing genes involved in “cellular process” (8384) and “metabolic process” (7134). In the cellular component category, most genes were involved in “cellular anatomical entity” (6784), “intracellular” (3769), and “protein-containing complex” (2864). The molecular function category included “binding” (7627) and “catalytic activity” (5665). Figure 3 presents the eukaryotic orthologous group (KOG) classification of the experimental unigenes which belonged to 26 different functional categories. The KEGG annotation of the experimental transcripts in the hepatopancreas of F-shrimp and S-shrimp showed that the annotated unigenes were categorised into five major pathways of KEGG databases: cellular processes, environmental information processing, genetic information processing, metabolism, and organismal systems (Fig. 4).

### DEG and real-time RT-PCR analysis

**Figure 5:**
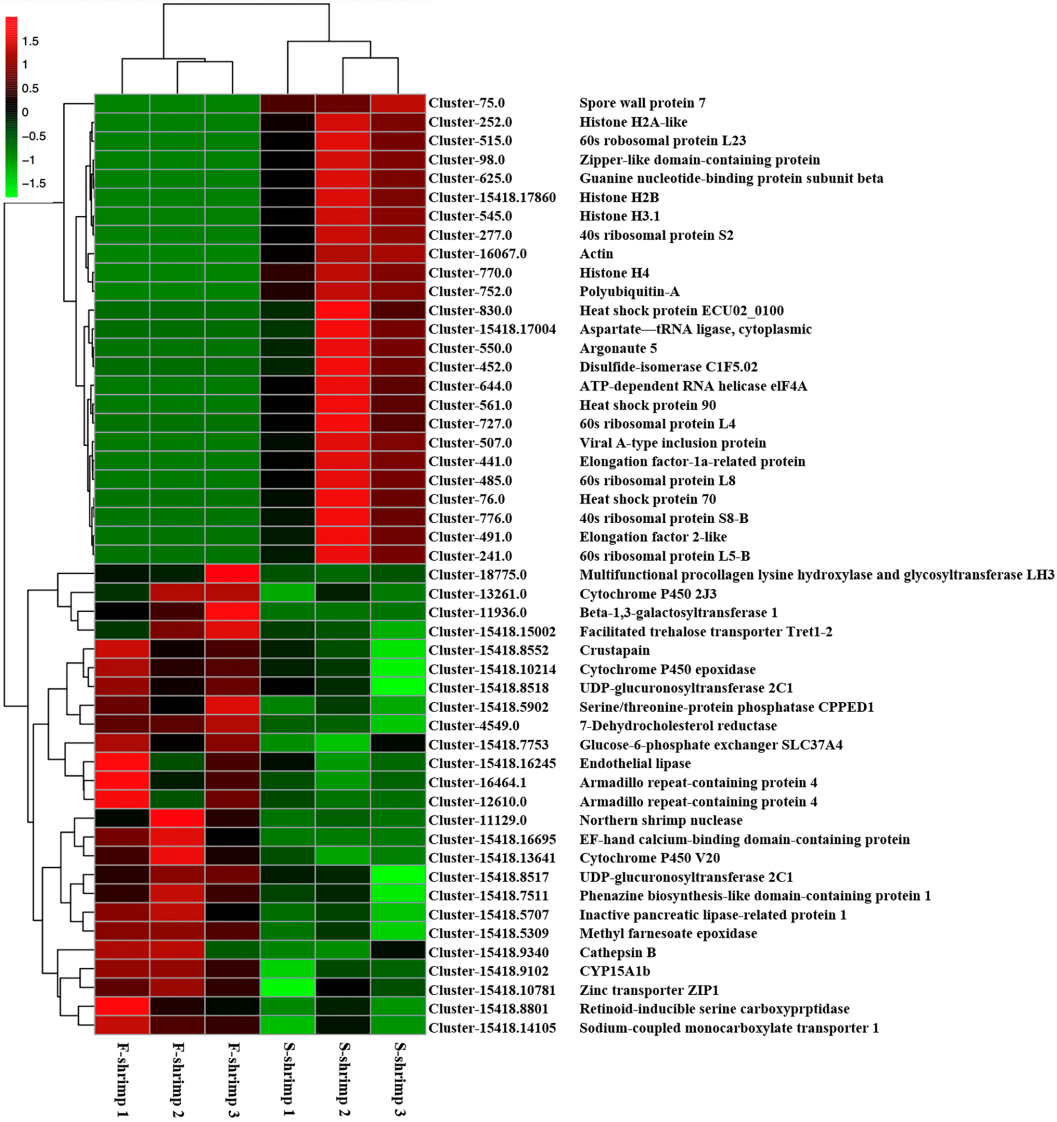
Heatmap plots for differentially expressed transcripts between fast-growing shrimp (F-shrimp) and slow-growing shrimp (S-shrimp). Red denotes genes with high expression levels, and green denotes genes with low expression levels. The colours ranging from red to green represent the log10 (FPKM + 1) value from large to small. Only the top 50 DEGs were used to construct the heatmap. The plots were conducted using Plotting tools: R (Version 3.0.3) ggplot2 package, pheatmap package (https://ap-magic.novogene.com/customer/main#/visitor-main/heatmap).

**Figure 6:**
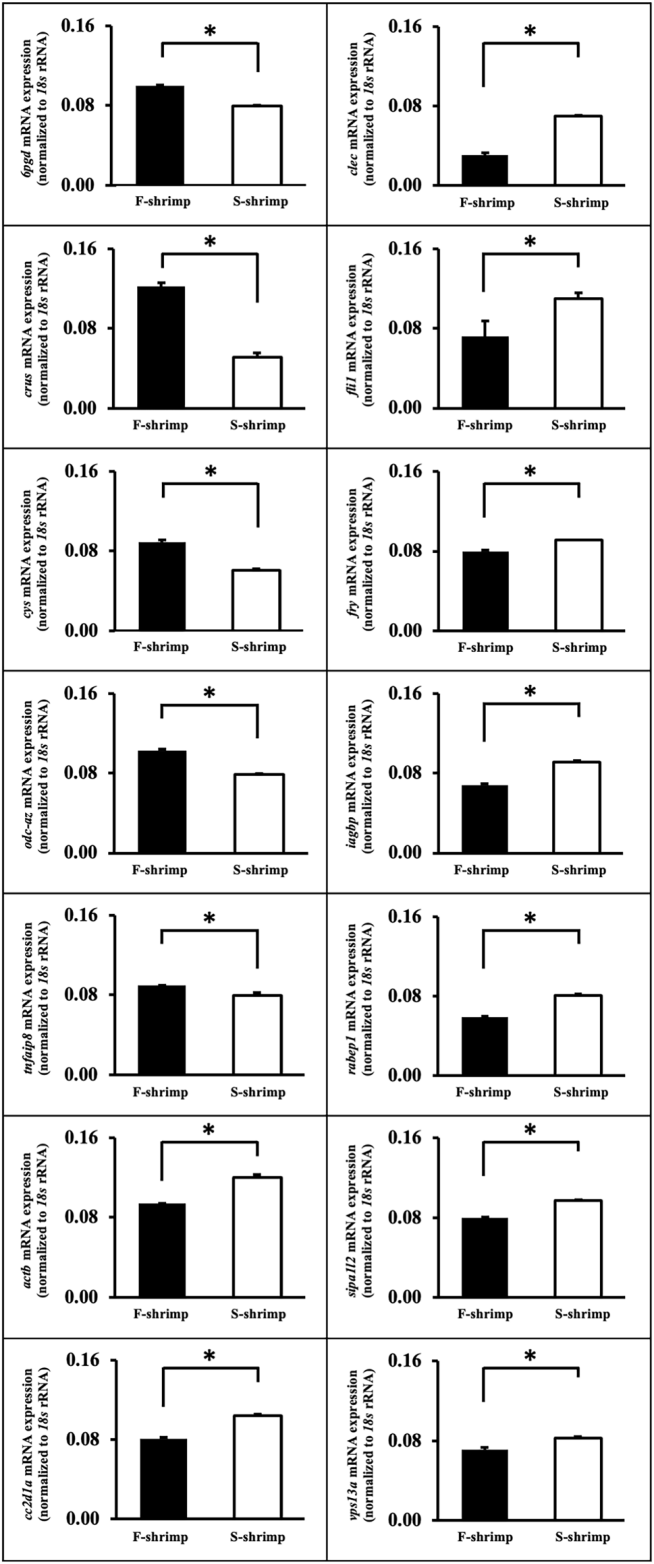
Differential expression of selected genes between fast-growing shrimp (F-shrimp) and slow-growing shrimp (S-shrimp) using qRT-PCR analysis. Selected genes that were crucial in physiologic and metabolic processes including crustapain (*cys*), crustin (*crus*), ornithine decarboxylase antizyme-1 (*odc-az*), 6-phosphogluconate dehydrogenase (*6pgd*), actin cytoplasmic 1 (*actb*), tumor necrosis factor alpha-induced protein 8 (*tnfaip8*), coiled-coil and C2 domain-containing protein 1A (*cc2d1a*), furry protein (*fry*), C-type lectin 1 (*clec*), insulin-like androgenic gland hormone binding protein (*iagbp*), signal-induced proliferation-associated 1-like protein 2 isoform X2 (*sipa1l2*), vacuolar protein sorting-associated protein 13A-like isoform X2 (*vps13a*), Rab GTPase-binding effector protein 1 (*rabep1*), and friend leukemia integration 1 transcription factor (*fli1*) have been shown.

Our study focused on DEGs with a log_2_ fold change > 1 and q-value < 0.05. Figure S4 shows the volcano plot of the DEGS between F-shrimp and S-shrimp. Figure 5 showed Heatmap plots for differentially expressed transcripts that presents clustering of top 50 DEGs in hepatopancrease between F-shrimp and S-shrimp. The results showed that 531 unigenes were upregulated and downregulated in F-shrimp compared to those in S-shrimp (Table S2). Table 7 shows the top 10 genes that were upregulated in F-shrimp and S-shrimp when compared with S-shrimp and F-shrimp, respectively. In addition, based on RNA sequence information obtained in this study, quantification of expression was carried out for several genes that are essential for metabolic and immune processes. Differential expression of *6pgd, crus, cys, odc-az, actb, tnfaip8, cc2d1a, clec, fli1, fry, iagbp, rabep1, sipa1l2* and *vps13a* were determined using qRT-PCR analysis (Fig. 6).

**Table 7:** Top 10 DEGs between F-shrimp and S-shrimp.

Gene name	Gene description	Gene ID	Shrimp demonstrating upregulation	Log_2_ fold change
*b3galt1*	Beta-1,3-galactosyltransferase 1-like	Cluster-11936.0	F-shrimp	7.8611
*efcab1*	EF-hand calcium-binding domain-containing protein 1	Cluster-15418.16695	F-shrimp	6.9352
*nuc1*	Northern shrimp nuclease	Cluster-11129.0	F-shrimp	5.8528
*plod3*	Multifunctional procollagen lysine hydroxylase and glycosyltransferase LH3-like	Cluster-18775.0	F-shrimp	5.1915
*armc4*	Armadillo repeat-containing protein 4	Cluster-16464.1	F-shrimp	3.5972
*cyp4v20*	Cytochrome P450 V20	Cluster-15418.13641	F-shrimp	3.1684
*risc*	Retinoid-inducible serine carboxypeptidase	Cluster-15418.8801	F-shrimp	3.0897
*smct1*	Sodium-coupled monocarboxylate transporter 1	Cluster-15418.14105	F-shrimp	2.9573
*pnliprp1*	Inactive pancreatic lipase-related protein 1	Cluster-15418.5707	F-shrimp	2.8515
*cyp15a1b*	CYP15A1b	Cluster-15418.9102	F-shrimp	2.7033
*ef1a*	Elongation factor-1a-related protein	Cluster-441.0	S-shrimp	9.9696
*hsp70*	Heat shock protein 70	Cluster-76.0	S-shrimp	9.3349
*act1*	Actin	Cluster-16067.0	S-shrimp	8.9102
*ago5*	Argonaute 5	Cluster-550.0	S-shrimp	8.3016
*ubq-1*	Polyubiquitin-A	Cluster-752.0	S-shrimp	7.9538
*rpl5b*	60S ribosomal protein L5-B	Cluster-241.0	S-shrimp	7.6765
*swp7*	Spore wall protein 7	Cluster-75.0	S-shrimp	7.6532
*rpl4*	60S ribosomal protein L4	Cluster-727.0	S-shrimp	7.5982
*dps1*	Aspartate–tRNA ligase, cytoplasmic	Cluster-15418.17004	S-shrimp	7.5654
*tif1*	ATP-dependent RNA helicase eIF4A	Cluster-644.0	S-shrimp	7.4893

*F-shrimp* fast-growing shrimp, *S-shrimp* slow-growing shrimp.

## Discussion

Plant-based ingredients have been the most commonly used to replace fishmeal in aquafeeds, and soybean meal has been the most common plant-based protein ingredient used to replace fishmeal in shrimp diets. The effects of SBM as a dietary protein source to replace FM in shrimps on growth performance varies depending on the shrimp species, level of replacement, and growth phases. For example, replacement of FM with SBM at 25–100% in the Pacific white shrimp during the juvenile stage led to reduced growth performance^9,12,13^. However, total replacement of FM with SBM was possible in the diet of Pacific white shrimp without adverse effects on growth performance^10,14^. Overall, in addition to these variable effects of the SBM inclusion diet (replacement of FM), the possibility that the growth response of shrimp might depend on the shrimp individual could not be excluded. Therefore, in this study, we selected shrimp according to differences in SGR, including high SGR (F-shrimp) and low SGR (S-shrimp), to investigate differences in body composition and haemolymph chemistry parameters. In addition, high-throughput RNA-seq technology was used to investigate DEGs which could be used to interpret the differences in metabolic and physiological responses between F-shrimp and S-shrimp.

Generally, whole-body chemical and/or carcase composition and plasma chemistry have been used to interpret the metabolic responses related to the nutritional status of fish^38,39^. For example, with similar means of growth response, Pacific white shrimp fed different levels of SBM inclusion diet showed no significant changes in haemolymph glucose and protein contents and whole-body protein composition^10^. In addition, pacific white shrimp fed a total replacement SBM-based diet which exhibited a lower average growth response, showed no significant changes in body composition compared to shrimp fed a non- or partial replacement SBM-based diet^9^. In this study, among carcass composition (crude protein, crude lipid, ash) as well as haemolymph chemistry (glucose, triglyceride, cholesterol, protein, HUN, AST, ALT, Mg, Ca, and P), we found that fast-growing shrimp had higher protein content in the carcasses than slow-growing shrimp. Our findings suggest higher muscle protein anabolism in F-shrimp which might reflect the differences in metabolism between fast-growing and slow-growing shrimp individuals.

Transcriptomic analysis was conducted to investigate the differences in physiological and metabolic responses at the molecular level between F-shrimp and S-shrimp fed an SBM-based diet. For a comparative study of distinct growth responses, SGRs that were two times higher in fast-growing shrimp and slow-growing shrimp were selected. In shrimp, the hepatopancreas is a major organ for metabolism, including the digestion, absorption, and storage of nutrients which play important roles in growth and a number of physiological processes. Therefore, different metabolic and physiological responses between F-shrimp and S-shrimp related to a specific diet should be used to underline the mechanism of efficient use of specific nutrients. Our results showed that 44,088 unigenes were obtained after the assembly. Among these assembled unigenes, 19,518 (44.27%) and 1857 (4.21%) of them were successfully annotated to at least one database and all databases, respectively. Depending on the output size and experiments, the total unigenes obtained from the hepatopancreas varied among shrimp species, including 29,180 unigenes in *Marsupeneaus japonicus*^21^, 63,453 unigenes in *Macrobrachium nipponense*^22^, 10,425 unigenes in *L. vannamei*^40^ and 106,644 unigenes in *L. Vannamei*^19^. The transcriptome of hepatopancreas in *M. nipponense* revealed that 2311 unigenes (3.64%) of assembled unigenes were matched to all databases, and of 5235 unigenes (5.05%) in *L. vannamei* were annotated in all databases^19^. Taken together, these findings suggest that compared to other shrimp species, a high number of annotated genes were obtained in *L. vannamei* because *L. vannamei* has been widely investigated using bioinformatics, leading to a high number of annotated genes. In addition, GO, KEGG, and KOG classifications demonstrated that the most expressed genes were similar to those reported in previous studies^20–22,19^. Our RNA informatics provides transcript sequences for several gene of interest in metabolic and physiologic processes which were further validated using qRT-PCR. Although qRT-PCR was more sensitive to evaluate differential level of gene expression, most results (except for *tnfaip8, rabep1, 6pgd*) of DEGs by RNA seq were consistent with qRT-PCR, demonstrating that RNA-seq technology could be a useful tool to explore possible DEGs. Overall, by means of assembled unigene numbers and functional classification, our transcriptome sequencing and annotation were of considerably good quality and informative for further determination of global DEGs.

Hepatopancreas is the main storage organ of key nutrients for proper growth and development^41^; therefore, using RNA-seq analysis and qRT-PCR, DEGs could be used to suggest an overview of physiologic and metabolic processes between fast- and slow-growing shrimp individuals. Overall, our findings revealed that several genes encoding physiological and metabolic enzymes showed upregulated expression in fast-growing shrimp compared to slow-growing shrimp, suggesting that these enzymes/proteins might directly and/or indirectly mediate the efficient use of SBM-based diets for growth promotion in shrimp individuals.

For example, beta-1,3-galactosyltransferase (*b3galt1*) is crucial for the transfer of uridine diphosphate galactose (UDP-galactose) which is an intermediate in polysaccharide production, to galactose with a terminal beta-N-acetylglucosamine residue. It is also involved in the biosynthesis of N-glycans which are essential for tissue structure, integrity, and energy storage^42^. Procollagen lysine hydroxylase and glycosyltransferase (*plod3*) which encode lysyl hydroxylase 3 (LH3), play an important role in the formation of collagen cross-links during collagen biosynthesis. Hydroxylation of lysyl and O-glycosylation of hydroxylysyl residues produces galactose or disaccharide (glucose-galactose) derivatives which are crucial in the posttranslational modification of collagen. Consequently, mutation of *plod3* led to disorders of connective tissue^43^. The enzyme, 6-phosphogluconate dehydrogenase (*6pgd*), plays an important role in the oxidative pentose phosphate pathway. It is involved in the conversion of 6-phosphogluconate to ribulose-5-phosphate, a ribose precursor for the synthesis of nucleotides and nucleic acids. It has also been found to be related to nicotinamide adenine dinucleotide phosphate (NADPH) generation in carbohydrate and lipid metabolism. For instance, increased dietary lipids enhanced the activity of 6PDGH in yellowtail snapper (*Ocyurus chrysurus*^44^. Dietary carbohydrate supplementation leads to increased 6PGDH activity in *Penaeus monodon*^45^. Genes encoding proteins and nucleic acid metabolism were upregulated in F-shrimp fed an SBM-based diet. For instance, nuclease (*nuc1*), an enzyme capable of cleaving or degrading nucleic acids, has been cloned and characterised from the hepatopancreas of shrimp^46^. In shrimp, proteins are the main energy source^47^. Retinoid-inducible serine carboxypeptidase1 (*risc*), also known as *scpep1* is localised in the lysosomes and act protease activity by inducing retinoic acid^48^. Crustapain is a papain-like cysteine proteinase which is referred to as cathepsin. These enzyme families play a crucial role in lysosomal protein hydrolysis, which is involved in various vital processes in shrimp. Cathepsin L is involved in moulting, and its single nucleotide polymorphism is related to growth in various shrimp^49^. Ornithine decarboxylase (ODC) is an enzyme involved in polyamine biosynthesis and plays an important role in protein and nucleic acid synthesis^50^. Polyamines are synthesised from L-methionine and L-ornithine, which are produced during the urea cycle^51^. Ornithine decarboxylase antizyme 1 plays a key role in the degradation of ornithine decarboxylase which negatively regulates ODC function^52^.

Although proteins are the main energy source, lipids also supply energy via catabolism. Induction of genes related to lipid metabolism in fast-growing shrimp would suggest an increase in the utilisation of lipids as an energy source which might enable the efficient use of proteins for growth in shrimp individuals. Upregulation of pancreatic lipase-related protein 1 (*pnliprp1*) in fast-growing shrimp could suggest an elevation of lipase activity for lipid metabolism in shrimp fed SBM-based diets. Cytochrome P450 (*cyp4v20,cyp15a1b*) is involved in the regulation of lipid homeostasis and plays an important role in detoxification processes in vertebrate livers. In mice, linoleic acid mediates hepatic *p450* induction^53^. In addition, sodium-coupled monocarboxylate transporter (*smct1*) mediates the sodium-dependent transporter of monocarboxylates, such as short-chain fatty acids, which supply energy in energy metabolism. It has also been shown to mediate the transportation of methionine^54,55^. However, a lower expression of insulin-like androgenic gland hormone-binding protein (*iagbp*) was observed in fast-growing shrimp. Expression of *iagbp* is involved in the insulin androgenic gland pathway which plays an important role in eyestalk-testis pathways in male gonad development. Its expression and function in the hepatopancreas were suggested to be involved in carbohydrate metabolism in the blue crab *Callinectes sapidus*, revealing its function like insulin in vertebrates^56^. It could be hypothesised that because proteins are generally superior to carbohydrates as an energy source in shrimp, downregulation of *iagbp* was observed in fast-growing shrimp. Taken together, these findings suggested that up-regulation of the expression of several genes related to energy metabolism would contribute effective utilization of SBM-based diets in fast-growing shrimp.

Several DEGS which were related to the immune system were upregulated in fast-growing shrimp. For example, *armc4*, *efcab1*, *crus* and *tnfaip8* are upregulated in F-shrimp. Armadillo repeat-containing protein 4 (*armc4*) encodes the armadillo repeat domain which is involved in the expression of antimicrobial peptides in the immune system. Interaction of hemocyanin via its ARM repeat domain with MKK4 promoted the transcription of genes expressing antimicrobial peptides^57^. The EF-hand calcium-binding domain-containing protein (*efcab1*) encodes calcium-responsive proteins which are involved in various cellular functions. In disk abalone, induction of expression of the EF hand domain-containing calcium-regulatory gene followed by bacterial infection suggests its role in immunity^58^. Curstin (*crus*) is an antimicrobial peptide which plays an important role in the innate immune mechanisms of shrimp^59^. Tumor necrosis factor alpha (*tnf*) activates the innate immune system and promotes inflammatory responses^60^. Tumour necrosis factor (TNF) has been reported to be involved in innate immune defense in kuruma shrimp (*Marsupenaeus japonicas*), although it has not been detected in the hepatopancreas^61^.

Alternatively, several genes were more highly expressed in slow-growing shrimp, suggesting different immune processes between fast- and slow-growing shrimp individuals. Expressions of the genes *clec*, *mbnl3*, *rabep1*, *vps13a*, *sipail*2, *ubq-1*, and *swp7* were upregulated in slow-growing shrimp. For instance, C-type lectins have been demonstrated to be involved in innate immune processes in shrimp^62^. Mucin plays an important role in mucosal immunity^63^. Dietary amino acids, particularly threonine, have been shown to modulate intestinal mucin levels. For example, inappropriate threonine levels lead to increased mucin synthesis^64^. Higher expression of proteins associated with phagocytosis and autophagy was found in slow-growing shrimp fed a plant-based diet. Rab GTPase-binding effector protein 1 (RABEP1; Rabaptin-5) acts as an effector of the GTPase Rab5. Expression of *rabep1* is associated with endocytosis, and its protein is related to the phagocytosis process^65^. In addition, vacuolar protein sorting associated protein 13 A (*vps13a*) was reported to be associated with phagosomes and plays important roles in efficient phagocytosis^66^. Signal-induced proliferation associated 1 like protein 2 (*Sipail2*), a Rap GTPase-activating protein, has been reported to be involved in amphiosomes in the autophagic pathway. Argonaute 5 (ago5) belongs to the (ago) gene family which plays an essential role in RNA degradation by silencing gene expression as well as antiviral function^67^. The ubiquitin proteasome pathway of protein degradation is important for immune modulation in shrimp, and increased expression of ubiquitin has been reported in shrimp infected with white spot syndrome virus^68^. Furthermore, spore wall protein has been demonstrated to be a marker for hepatopancreatic microsporidiosis which was reported to be a cause of growth retardation in shrimp farming^69^. Combined together, upregulation of several immune-related genes could interpret trade-offs between growth and immunocompetence in these expressed genes.

While fast-growing shrimp exhibit high quantity of transcripts that are involved in metabolic and physiologic processes, slow-growing shrimp had higher expression of several genes that are categorized in housekeeping genes including *fli1, cc2d1a, ef1a, hsp70, rpl5b, rpl4, dps1*, *tif1*, *act1*, *actb, fry* and *hsp70*. In general, the expression of housekeeping genes is vital for the maintenance of various basic cellular functions. For example, coiled-coil and C2 domain-containing protein1A (*cc2d1a*) have been reported to regulate the transcription of several genes. For example, it has been demonstrated to act as a transcriptional repressor of dopamine and serotonin receptor genes, and its expression has been shown to be involved in diverse physiological functions^70^. Friend leukaemia integration 1 (*fli1*) is a transcription factor involved in diverse physiological processes^71^. In addition, *ef1a1, rpl5b, rpl4, dps1*, and *tif1* encode proteins and/or enzymes that are essential for protein biosynthesis, such as components of ribosomes, translation initiation by aiding mRNA binding to ribosomes, ligation of tRNA with their relative amino acids, and translation elongation proteins^72^. Moreover, cytoskeletal proteins such as actin (*actb*) have been demonstrated to be important in many biological processes, and furry protein (*fry*) has been reported to be important in regulating actin cytoskeleton expression^73^. In aquatic animals, health and nutritional status modulate the expression of actin. For example, *L*. *vannamei* infected with *Vibrio parahaemolyticus* and WSSV led to decrease actin expression^74^. Heat shock protein 70 (*hsp70*) which is also categorised as a housekeeping gene, was shown to be upregulated in response to disease resistance^75^.

## Conclusions

In conclusion, a higher muscular protein composition suggests protein anabolism in fast-growing shrimp individuals. Using RNA seq and/or qRT-PCR, in fast-growing shrimp, upregulation of genes associated with physiologic and metabolic enzymes/proteins was found while a high quantity of housekeeping gene transcripts was observed in slow-growing shrimp. Different DEGs related to immune mechanisms were found in fast- and slow-growing shrimp individuals, demonstrating different immunocompetence associated with growth.

### Supplementary Information

Supplementary Figure S1.

Supplementary Figure S2.

Supplementary Figure S3.

Supplementary Figure S4.

Supplementary Table S1.

Supplementary Table S2.

## Data Availability

All data supporting the results of this study are available withing the manuscript and the supplementary files. The RNA sequencing data are available in SRA database, accessions PRJNA940791, https://www.ncbi.nlm.nih.gov/sra/PRJNA940791.
