# Supplementary material for: Differential responses of hepatopancreas transcriptome between fast and slow growth in giant freshwater prawns (Macrobrachium rosenbergii) fed a plant-based diet
Source: Sci Rep. 2024 Feb 29;14:4957. doi: 10.1038/s41598-024-54349-6 (PMC10902295; doi:10.1038/s41598-024-54349-6)
Supplement: Supplementary file 4 — Supplementary Figure S4. [file 41598_2024_54349_MOESM4_ESM.docx]

**Supplementary information**

**Differential responses of hepatopancreas transcriptome between fast and slow growth in giant freshwater prawns (*Macrobrachium rosenbergii*) fed a plant-based diet**

Khanakorn Phonsiri^1^, Rapeepat Mavichak^2^, Stephane Panserat^3^, Surintorn Boonanuntanasarn^1,*^

^1^School of Animal Technology and Innovation, Institute of Agricultural Technology, Suranaree University of Technology, 111 University Avenue, Muang, Nakhon Ratchasima, 30000 Thailand

^2^Aquatic Animal Health Research Center, Charoen Pokphand Co. Ltd., Rama 2 Rd., Km 41.5, T. Bangtorat, Muang Samutsakorn, Samutsakorn 74000, Thailand

^3^INRAE, Université de Pau et des Pays de l'Adour, E2S UPPA, NuMéA, Saint-Pée-sur-Nivelle, France

*Corresponding author:

S. Boonanuntanasarn; surinton@sut.ac.th, Tel: +6644224371, Fax: +6644224150


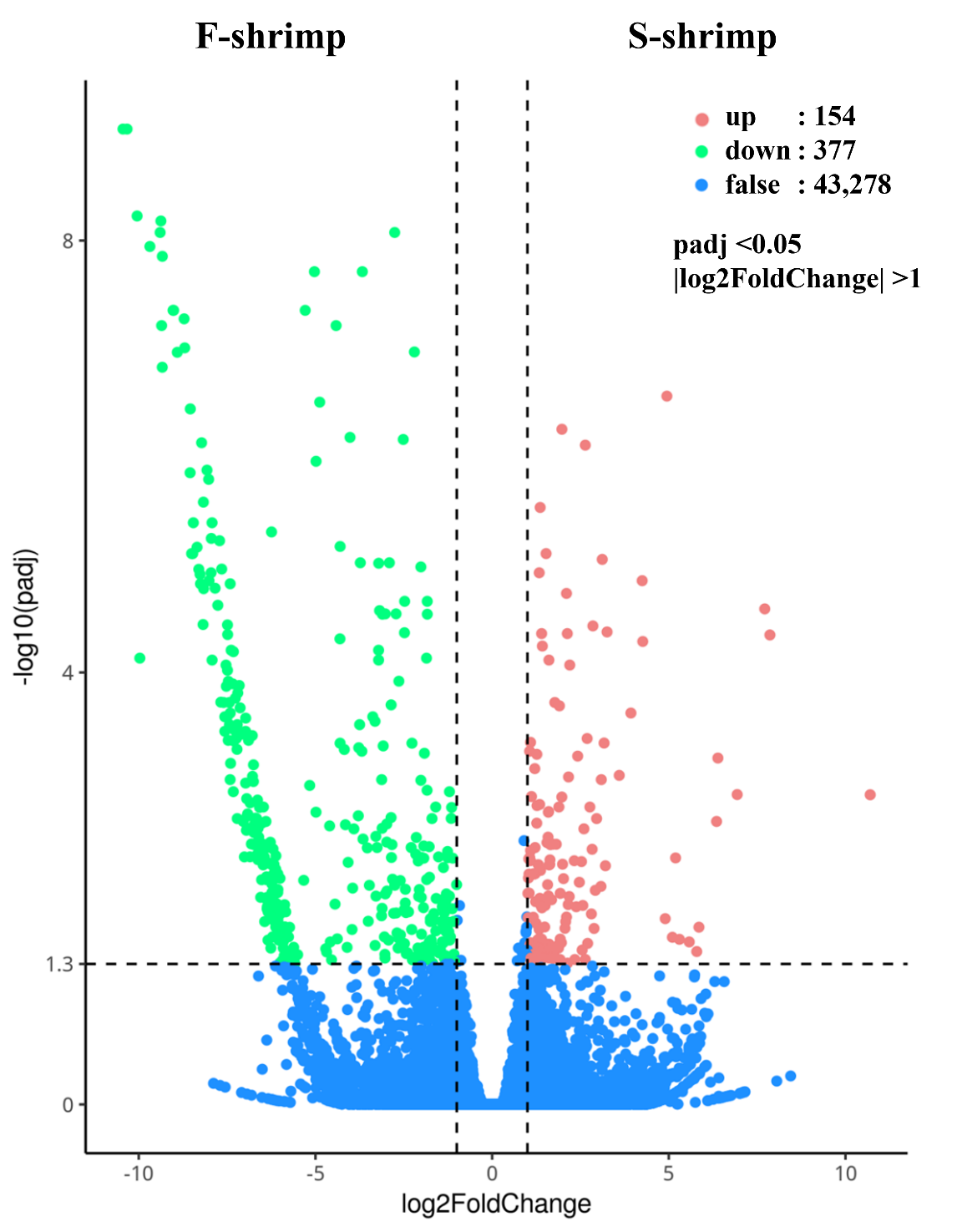


Figure S4. Volcano plot displaying DEGs identified between fast-growing shrimp (F-shrimp) and slow-growing shrimp (S-shrimp). Significantly upregulated and downregulated expressed genes in fast-growing shrimp compared with slow-growing shrimp (padj <0.05, log2FoldChange >1) are denoted as pink and green dots, respectively. The X-axis represents the fold change in gene expression, and the Y-axis demonstrated the significant differences.
