# Supplementary material for: Differential responses of hepatopancreas transcriptome between fast and slow growth in giant freshwater prawns (Macrobrachium rosenbergii) fed a plant-based diet
Source: Sci Rep. 2024 Feb 29;14:4957. doi: 10.1038/s41598-024-54349-6 (PMC10902295; doi:10.1038/s41598-024-54349-6)
Supplement: Supplementary file 5 — Supplementary Table S1. [file 41598_2024_54349_MOESM5_ESM.docx]

**Supplementary information**

**Differential responses of hepatopancreas transcriptome between fast and slow growth in giant freshwater prawns (*Macrobrachium rosenbergii*) fed a plant-based diet**

Khanakorn Phonsiri^1^, Rapeepat Mavichak^2^, Stephane Panserat^3^, Surintorn Boonanuntanasarn^1,*^

^1^School of Animal Technology and Innovation, Institute of Agricultural Technology, Suranaree University of Technology, 111 University Avenue, Muang, Nakhon Ratchasima, 30000 Thailand

^2^Aquatic Animal Health Research Center, Charoen Pokphand Co. Ltd., Rama 2 Rd., Km 41.5, T. Bangtorat, Muang Samutsakorn, Samutsakorn 74000, Thailand

^3^INRAE, Université de Pau et des Pays de l'Adour, E2S UPPA, NuMéA, Saint-Pée-sur-Nivelle, France

*Corresponding author:

S. Boonanuntanasarn; surinton@sut.ac.th, Tel: +6644224371, Fax: +6644224150

**Table S1** Primers used for qRT-PCR and the expected size of the amplicons

| **Genes** | **Sequence (5’-3’)** | | **Expected size (bp.)** |
| --- | --- | --- | --- |
| *18s* | Forward | GAGACGGCTACCACATCTAAG | 182 |
|  | Reverse | ATACGCTAGTGGAGCTGGA |  |
| *6pgd* | Forward | TGAGGTGAGCAGTTGCCTTT | 162 |
|  | Reverse | TGCAGAGCACTTGTAGGACT |  |
| *actb* | Forward | AAGGGTACGCCTTACCTCAC | 213 |
|  | Reverse | TCTCGAGGGAGGATGATTGC |  |
| *cc2d1a* | Forward | GGCTCTTGCTCGTGTCTTTA | 108 |
|  | Reverse | ACCAGTGGAACCTTCACAGA |  |
| *clec* | Forward | GCGTCAGAATGCACACTCC | 181 |
|  | Reverse | CCGGATTTCCTGTTCTGGTG |  |
| *crus* | Forward | CATCCACGCACTCTTGATCC | 232 |
|  | Reverse | AGCACCAACATGAGATTCGC |  |
| *fli1* | Forward | CCTCGACTACATGCTGGGAT | 262 |
|  | Reverse | GGGCTCCAGGATCTTGAACT |  |
| *fry* | Forward | AACTAGTGGCAGACCAACGA | 214 |
|  | Reverse | TCTGTCAGACGGAAGCTTGT |  |
| *iagbp* | Forward | CTAGAGATCCAGGACTCGCA | 101 |
|  | Reverse | GGGTGCATTCCAGGGTAGAT |  |
| *cys* | Forward | CCTTCAAGAAGCCGTCCAAG | 195 |
|  | Reverse | CAGGAGTTCCTGACGATCCA |  |
| *odc-az* | Forward | AGTTGGCGGAACAAACAACA | 217 |
|  | Reverse | AACCCGAGGAACATGAAGGT |  |
| *rabep1* | Forward | GCAGCACGAAGAAGATCAGG | 231 |
|  | Reverse | GGTGAATGTGGAGGCTCAGA |  |
| *sipall2* | Forward | ACGGTTACCTGGACAAGAGG | 214 |
|  | Reverse | CTTTCTGGGTGTTGCAGCTT |  |
| *tnfaip8* | Forward | GGAGGATAGACCACGTCCTG | 125 |
|  | Reverse | GTCGAAGGCTTTGCTCATGT |  |
| *vps13a* | Forward | GCCTTACAACAGCCATGAGG | 227 |
|  | Reverse | GCTGACGGAGAAGATTACGC |  |

*18s*; 18s ribosomal RNA, *6gpd*; 6-phosphogluconate dehydrogenase, *actb*; Actin cytoplasmic 1 isoform X2, *cc2d1a*; Coiled-coil and C2 domain-containing protein 1, *clec*; C-type lectin, *crus*; Crustin, *fli1*; Friend leukemia integration 1, *fry*; Protein furry-like, *iagbp*; Insulin like androgenic gland hormone binding protein, *cys*; Crustapain, *odc-az*; Ornithine decarboxylase antizyme 1, *rabep1*; Rab-GTPase-binding effector protein 1, *sipa1l2*; Signal-induced proliferation-associated 1 like protein 2 isoform X2, *tnfaip8*; Tumor necrosis factor alpha-induced protein 8 like protein, *vps13a*; Vacuolar protein sorting-associated protein 13A like isoform X2.
